# Coping with COVID: Developing a Rapid-cycle Frontline Quality-improvement Process to Support Employee Well-being and Drive Institutional Responsiveness in a Tertiary Care Faith-based Hospital in Rural Kenya

**DOI:** 10.4269/ajtmh.20-1661

**Published:** 2021-06-15

**Authors:** Mary B. Adam, Naomi Wambui Makobu, Wilson Karuri Kamiru, Simon Mbugua, Faith Mailu

**Affiliations:** 1 AIC Kijabe Hospital, Kijabe, Kenya;; 2 Africa Consortium for Quality Improvement Research in Frontline Healthcare (ACQUIRE), Kijabe, Kenya

## Abstract

The coronavirus disease 2019 (COVID-19) pandemic has demanded rapid institutional responses to meet the needs of patients and employees in the face of a serious new disease. To support the well-being of frontline staff, a series of debriefing sessions was used to drive a rapid-cycle quality-improvement process. The goals were to confidentially determine personal coping strategies used by staff, provide an opportunity for staff cross-learning, identify what staff needed most, and provide a real-time feedback loop for decision-makers to create rapid changes to support staff safety and coping. Data were collected via sticky notes on flip charts to protect confidentiality. Management reviewed the data daily. Institutional responses to problems identified during debrief sessions were tracked, visualized, addressed, and shared with staff. More than 10% of staff participated over a 2-week period. Feedback influenced institutional decisions to improve staff schedules, transportation, and COVID-19 training.

## INTRODUCTION

The coronavirus disease 2019 (COVID-19) pandemic has demanded rapid institutional transitions to meet the needs of patients and employees facing a radical new disease.^1^ This has been an organizational challenge, especially in low- and middle-income countries, where the scarcity of essential supplies and already overstretched staff are an everyday reality. Hospitals delivering frontline care have had to find ways to face the organizational challenge of addressing employee well-being while delivering quality care to patients.

Obtaining knowledge of the perceived needs of employees in an environment where frontline staff fear for their own personal safety as well as job security is complicated. The COVID-19 pandemic has provided a “perfect storm” in Kenya, with its medical uncertainty while facing a novel infectious threat compounded by organizational disruption and financial distress.^2^ In Kenya, the health sector involves approximately 50% of health care services offered by government facilities and approximately 50% of services offered by private and/or faith-based facilities that have fee-for-service models.

Staff anxiety increased as salary cuts and furloughs were instituted to cope with the negative cash position in Kijabe created by the expense of personal protective equipment, reduced patient demand for services (secondary to recommendations to shelter in place), and the government-mandated disruption of elective surgery (a service with a higher profit margin). Many patients in Kijabe have lower incomes and are without health insurance. During the pandemic, barriers to routine care were further complicated by the increased COVID-19 exposure risk, increased public transportation costs, and restricted hours of travel related to strict curfews and various degrees of lockdown.

To support the well-being of frontline staff during this crisis and develop a confidential environment or “safe space” for employees to share their concerns, the Kijabe Maternal Newborn Community Health (MNCH) team proposed adapting a participatory technique (rooted in human-centered design) that they were using for their community work but had been curtailed by shelter-in-place restrictions.^3^^,^^4^ The hospital’s Quality Manager and Outpatient Director (in charge of frontline screening) embraced the idea because of the interest in supporting staff and identifying stress points that could be addressed by management. The decision was made to use a series of debrief sessions to form of a rapid-cycle quality-improvement process following a Plan-Do-Study-Act approach with ethics committee oversight involving the following objectives: determine personal coping strategies used by staff and provide an opportunity for staff cross-learning; ask staff about what they need most; and provide a real-time feedback loop for decision-makers to support staff while coping with and managing stress during the COVID-19 outbreak. We present our experience as a potential solution for others.

## METHODS

The quality-improvement process followed a qualitative study design with a focus group discussion format.

### Sampling and recruitment.

The human resources (HR) department sent an e-mail with a letter of invitation to all department heads notifying them of the debrief sessions and encouraging supervisors to make this opportunity available to staff during their paid work time. Invitations to frontline staff came in the form of e-mail from the HR department and personal invitations from the MNCH staff. Because the MNCH team members work “beyond the walls” of the hospital, they were a neutral face representing neither management nor hospital-based staff. Frontline managers, the HR department, and senior staff were excluded to ensure sufficient confidentiality; however, were offered other sessions separately.

### Setting.

Kijabe Hospital is a faith-based nonprofit hospital approximately 1.5 hours outside Nairobi, Kenya. It is a 350-bed facility that performs more than 10,000 surgical procedures and treats more than 130,000 outpatients each year. This study was performed 2 weeks after the first COVID-19 case was confirmed in Kenya.

### Ethics.

The Kijabe Hospital Ethics Review Committee approved the process and procedures. Support of the ethics committee was considered essential to guarantee confidentiality for staff members in case they had comments that were critical of the administration.

### Consent.

The consent process included an oral explanation of the purpose of the debrief sessions, what the participation involved, participant rights, measures for security and privacy, and the opportunity to ask for more information and feedback. All participants signed written informed consent. To assure confidentiality, sessions were not recorded.

**Figure 1.**
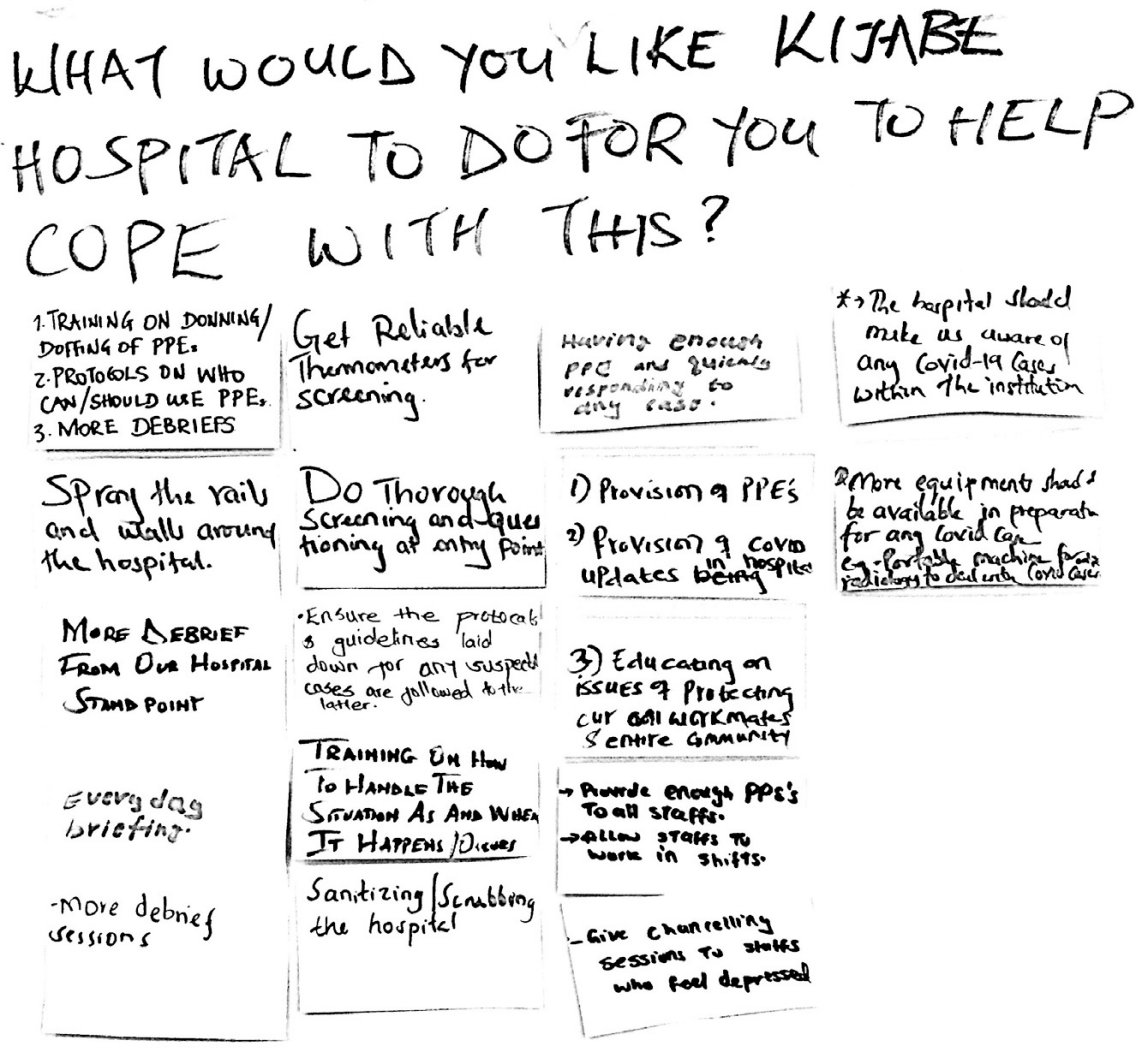
Sample of responses to the question, “What can Kijabe Hospital do for you?”

All focus group discussions were conducted in a large education room during work hours with approximately 5 to 10 participants seated 1.5 m apart. Each session lasted approximately 1.5 hours. Sessions occurred over a 2-week timeframe, thus allowing night shift personnel an opportunity to rotate to days to participate. Because hospital occupancy and outpatient volume were very low, allowing staff to attend did not pose a risk to patient care. No additional compensation was given for participation. A note-taker was present during every session, but data were primarily documented using sticky notes posted on a flip chart to preserve anonymity of the source (Figure 1).

Facilitators followed a discussion guide. After introductions, participants were encouraged to share how COVID-19 had changed their home and work environments using open-ended questions. After a period of sharing verbally, participants were encouraged to answer the following questions by writing their response on sticky notes:

1. What are some of the personal coping strategies you use?
2. What would you like Kijabe Hospital to do for you to help you cope with this?

Participants were given the opportunity to make changes to their sticky notes that were posted on a flip chart for all to see. At the end of the session, participants were able to confirm that their views were well-represented by the sticky notes as a form of data validation. At the end of the session, participants were asked to complete a three-item questionnaire to give facilitators feedback to improve the sessions.

### Data analysis.

The MNCH team met after each session. This allowed for an opportunity to refine the debrief session questions or process and to conducted a rapid synthesis of data from the session, including notes from the note-taker, observations, and the data provided on sticky notes. Managers reviewed the information from the sticky notes every day to identify factors that they could improve. The MNCH team tracked the management responses to staff issues and the date of the response. During the second phase of the analysis, the MNCH team sought to identify significant themes over time and across departments. The time element was important because the situation was constantly changing.

## RESULTS

During a 2-week period, 17 focus group discussions were conducted and more than 10% of all hospital staff (*N* = 122) voluntarily participated, representing a broad range of departments, such as Housekeeping, Housing, Kitchen, Nursing, Pharmacy, Laboratory, Radiology, Sewing, Nutrition, Physiotherapy, Finance, Clinical Officers, and Medical Officer Interns. More than 80% of the participants provided scores indicating that the sessions were valuable (1–10 Likert scale: 1, waste of time; 10, really valuable).

Responses to the question, “What are some of the personal coping strategies you use?” were shared on sticky notes. The five most popular personal coping strategies reported were prayer, reading the Bible, listening to music, watching movies, and interacting/sharing with family members.

Responses to the question, “What would you like Kijabe Hospital to do for you to help you cope with this?” were shared on sticky notes (Figure 1). These were reviewed every day by managers who met with the outbreak committee or relevant departments to determine what could be accomplished. Rapid-cycle management responses were tracked, visualized, and shared with staff.

By week 3, the demand for the sessions decreased and an inductive analysis of data regarding key themes was performed (Supplemental Table S1). Key themes were institutional preparedness, employee well-being, and COVID-19 risk reduction measures for family and visitors (and by proxy for staff).

## DISCUSSION

Debrief sessions allowed staff members to identify their own coping strategies, learn other coping strategies from colleagues, and share needs and concerns with management without fear. This rapid-cycle feedback loop facilitated management decision-making and priority-setting by allowing them to address employee issues that impacted employee well-being in real time. Issues that were addressed were removed from the list of ongoing staff concerns. Weak communication strategies indicated that staff members were often unaware of what management had performed to address concerns. In the face of a rapidly changing context, yesterday’s successes were somewhat diminished by the next day’s new challenges. The infographic (Supplemental Figure S1) was disseminated to system-wide WHATSAPP groups to reinforce management’s desire to listen, learn about staff concerns, and document progress to date.

This study had some limitations. Some departments were not represented during the exercise, including maintenance and chaplaincy. This could have occurred as a result of the timing of the debrief sessions and/or availability of these staff members. Thematic analyses using sticky notes without complete recordings and transcript analyses may have insufficiently represented the perspective of an individual participant; however, this was mitigated by the in-session participant validation of what was meant and by the recurring nature of the comments from across many departments, resulting in the saturation of themes.

## CONCLUSION

The debrief sessions were well-received and effective for providing anonymous feedback to management. Weak management communication strategies may undermine employee well-being and health system resilience under the stress of COVID-19. Rapid-cycle debrief cycles may be useful for other issues.

## References

[1] Lai J 2020. Factors associated with mental health outcomes among health care workers exposed to coronavirus disease 2019. JAMA Netw Open 3: e203976. 32202646 10.1001/jamanetworkopen.2020.3976 PMC7090843

[2] Khullar D Bond AM Schpero WL , 2020. COVID-19 and the financial health of US hospitals. JAMA 323: 2127–2128. 32364565 10.1001/jama.2020.6269

[3] Andresen M Potter T, 2017. Improving primary care with human-centered design and partnership-based leadership. *Interdisciplinary J Partnership Based Studies 4:* 10.24926/ijps.v4i2.166.

[4] Roberts JP Fisher TR Trowbridge MJ Bent C , 2016. A design thinking framework for healthcare management and innovation. Healthc (Amst) 4: 11–14. 27001093 10.1016/j.hjdsi.2015.12.002

